# Influence of Coal Mining on Historical Buildings: Case Study in Shanxi

**DOI:** 10.3390/ijerph20021543

**Published:** 2023-01-14

**Authors:** Yingfeng Sun, Shuaipeng Zhu, Zhiqian Peng, Chunran Yang, Biao Zhou, Xiaoliang Wang, Yixin Zhao

**Affiliations:** 1 Research Institute of Macro-Safety Science, University of Science and Technology Beijing, Beijing 100083, China; 2 School of Civil and Resource Engineering, University of Science and Technology Beijing, Beijing 100083, China; 3 School of Arts and Sciences, University of Pennsylvania, Philadelphia, PA 19104, USA; 4 School of Emergency Management and Safety Engineering, China University of Mining and Technology (Beijing), Beijing 100083, China; 5 Beijing Key Laboratory for Precise Mining of Intergrown Energy and Resources, China University of Mining and Technology (Beijing), Beijing 100083, China; 6 School of Energy and Mining Engineering, China University of Mining and Technology (Beijing), Beijing 100083, China

**Keywords:** coal mining, historic buildings, overlap, surface subsidence, Shanxi

## Abstract

Numerous historical buildings exist in Shanxi Province, a major coal producing area in China, so there exist many overlapping areas between ancient wooden buildings and coal mining. Coal mining in overlapping areas will lead to surface subsidence, which will have an impact on historical buildings. Based on the distribution of historical buildings and the distribution and mining of coal resources in Shanxi Province, this paper concludes that the overlapping areas of coal mining and ancient wooden buildings in Shanxi Province are mainly concentrated in Changzhi City, and the Lu’an mining area in Changzhi City is selected as the research object. In addition, using the gray correlation analysis method, the surface subsidence coefficient, which characterizes the intensity of mining subsidence, is used as the reference sequence. Seven factors selected from the geological conditions and mining conditions of the Lu’an mining area are used as the comparison sequence to calculate the gray correlation between each influencing factor and the surface subsidence coefficient, and to obtain that geological factors such as the nature of the overlying rock layer, bedrock thickness and dip angle of the coal seam, and mining factors such as mining height, average mining depth and working face size largely determine the surface subsidence coefficient. The surface subsidence in the overlap area could largely be influenced by geological factors such as the nature of the overlying rock layer, bedrock thickness and coal seam inclination, and mining factors such as mining height, average mining depth and working face size. Finally, we investigate the possible effects of surface subsidence on ancient wooden buildings in the overlapping area with the surface subsidence and formation mechanism and propose technical measures to reduce the effects of surface subsidence due to coal mining on historical buildings in the overlapping area.

## 1. Introduction

As the largest production and consumption of fossil energy in China, coal is the backbone of current industrial production and has formed a relatively complete and mature industrial chain. At present, because the technology of developing and utilizing new energy sources such as hydropower, wind energy, solar energy and ocean energy is not mature enough, it is impossible to solve the problem of being affected by climate and seasonal influences in the short term; coal is still the most reliable and abundant resource in China’s strategy and occupies an important position in the energy industry and national economy [1]. The energy endowment characteristic of “rich in coal, poor in oil and less in gas” fundamentally determines that China’s coal-based energy structure will not change for a long time. According to incomplete statistics, China’s total energy consumption in 2021 will be approximately 5.24 billion t of standard coal, of which coal consumption will account for approximately 56.0%.

While promoting rapid regional economic development, coal mining has also caused great damage and incalculable losses to the local ecological environment, the most prominent manifestation of which is surface subsidence. The primary task of surface subsidence research is to monitor the ground subsidence with high precision, high efficiency and periodicity [2] and to obtain a more realistic surface subsidence law as far as possible. The traditional monitoring methods such as level survey, total station and GPS (Global Positioning System) survey are only point-specific measurements, which are difficult to carry out in real time, dynamically, over a large area, and the monitoring cost is high [3,4,5]. The InSAR (Interferometry Synthetic Aperture Radar) monitoring method monitors ground subsidence by the phase difference of SAR images in transit [6], which presents the advantages of all-day use, wide range, low cost and high precision [7].

In recent years, domestic and foreign scholars have produced a large number of research results on the surface subsidence problem. Tang et al. used SBAS-InSAR technology to continuously monitor the surface deformation of Dongping coal mine in Shanxi Province from 30 May 2017 to 25 January 2018, and proved that the short-time revisited SAR data can accurately invert the surface subsidence rate and accumulation, and dynamically extract from the mine the spatial distribution and time variation process of the rapid deformation field [8]; Giardina et al. concluded that one of the important causes of structural damage of historical buildings is the ground subsidence caused by excavation works, and then conducted a 3D finite element analysis of soft soil tunnel excavation under masonry buildings [9]; Johnson indicated that evaporites are present in 32 of the 48 contiguous states of the United States, and they underlie approximately 35–40% of the land area. Karst is known at least locally (and sometimes quite extensively) in almost all areas underlain by evaporites, and some of these karst features involve significant subsidence [10]; Lee et al. dealt with the characteristics of subsidence and the relationships between the subsidence factors over abandoned coal mines in South Korea. The subsidence factors that were investigated were the dip angle and thickness of the coal bed, mining depth, depth of subsidence and subsidence area in 548 cases of subsidence that occurred throughout the country [11]; Solarski et al. used the case study of Bytom in southern Poland to evaluate the impact of multiple years of underground mining of minerals on changes in the elevation of an urban area, and concluded that the rate of anthropogenic subsidence in the city between 1883 and 2011 averaged 43 mm/year (5.5 m over the study period) [12]; Marschalko et al. identified the mutual connections between mined-out panels of a deposit and the final manifestations on the ground surface related to deep black coal mining [13]; Dang et al. applied the Persistent Scatterer Interferometric Synthetic Aperture Radar (PSInSAR) approach to 56 Sentinel-1A images, captured from June 2015 to June 2019 in order to map the spatial distribution of this phenomenon and monitor its evolution over the time [14]; Can et al. identified the effects of mining subsidence on masonry buildings in the mining area of Kozlu, Zonguldak, Turkey, and illustrated them with selected images of damaged masonry buildings [15]; Zhang Qing et al. established a model of the interaction between the building foundation and the ground, and calculated the change mechanism of the additional stress of the surrounding buildings caused by the excavation of the foundation pit and the coordination of the building with the ground by choosing a reasonable foundation subsidence equation or using the actual measured subsidence data [16]; Unlu et al. used a new surface subsidence prediction method (ISP-Tech technology) to obtain geological cross-sections using geological information collected from GIS and MIS systems to build a finite element model grid, and then performed a two-dimensional finite element simulation analysis of the ground subsidence caused by mining, and compared the ground subsidence predicted by the simulation study with GPS and D-InSAR technology measurements, successfully demonstrating that the ISP-Tech technology can be applied to complex mine subsidence problems with higher prediction accuracy [17]; Ma et al., in order to investigate the effect of surface subsidence in mining areas on surface buildings, studied reinforced concrete frame structures with different differential subsidence types using SAP2000 software, ultimately showing that damage caused by uneven subsidence will inevitably reduce the seismic performance of buildings in subsidence areas [18]; Zhou Junjie established a three-dimensional finite element calculation model of “tunnel-soil-ancient building” and investigated the current status of ancient buildings such as the small building at Tianxiang, the Confucian Temple and the North Temple tower and analyzed the geological data of the site, and then evaluated the impact of surface subsidence caused by the shield construction on the above three ancient buildings [19]; Song et al. used gray correlation analysis to analyze the relationship between the maximum surface subsidence coefficient and its influencing factors, indicating that the overlying rock thickness, disturbance coefficient and mining thickness ratio are the key factors affecting mining subsidence [20]; Zhang Meng et al. studied the No. 3 coal seam under the buildings in Lu’an area, and obtained five types of surface subsidence by constructing a fuzzy extension model of surface subsidence for paste backfill mining, and indicated that the overburden structure, the top slab sinking before backfill, the strength of the backfill and backfill technology are the key factors controlling surface subsidence [21]; Luan et al. selected eight influencing factors such as mining thickness, coal seam inclination, average mining depth, strike width-to-depth ratio, tendency width-to-depth ratio, advancement speed, Loose Strata thickness and overburden average solidity coefficient, etc., and used a combination of gray correlation analysis and principal component analysis to obtain the combined weights of the influencing factors of surface subsidence coefficient, and ranked the influencing factors of surface subsidence coefficient according to the combined weights to obtain the main influencing factors of surface subsidence coefficient. They determined the main influencing factors of surface subsidence coefficient, and then proposed a BP neural network model for prediction analysis of surface subsidence coefficient, and finally concluded that the combined weights of Loose Strata thickness, advancement speed, average mining depth and tendency width–depth ratio are greater and are the main influencing factors of surface subsidence coefficient [22]; Zhu et al. proved that the transition zone of hard soil or soft rock has a certain support effect on overlying rock and has a certain restraining effect on surface subsidence during mining, while the thickness ratio of Loose Strata, bedrock and transition zone will affect the degree of this restraining effect. The sensitivity ranking order of bedrock > transition > Loose Strata was obtained by neural network for the three factors [23]; Huang Cenli showed that the ground subsidence caused by coal mining is influenced by the mining depth, thickness, mechanical properties of the overburden, mining method, mining intensity and mining range, in addition to geological structure, topography and hydrogeological conditions [24].

In coal mining activities, every 10,000 tons of coal mined causes 400~2800 m^2^ of surface subsidence [25]. According to incomplete statistics, the total area of surface subsidence caused by coal mining in Shanxi Province has reached 6500 km^2^ [26]. The surface subsidence caused by coal mining in the province is mainly concentrated in Datong, Changzhi, Jincheng, Lvliang, Jinzhong and Linfen cities, accounting for more than 70% of the total area of surface subsidence caused by coal mining in the province. Two cities, Linfen and Lyuliang, have the largest area of surface subsidence due to coal mining, and Yuncheng city has the smallest area of surface subsidence due to coal mining.

As an essential coal production base in China and the province with the most significant number of existing ancient wooden buildings, there are many areas where coal mining and ancient buildings overlap in Shanxi. In this context, long-term coal mining has seriously damaged the geological structure of Shanxi Province and led to the increasingly prominent problem of surface subsidence in Shanxi Province, which has not only indirectly caused irreparable damage to Shanxi’s ancient wooden buildings but also brought an urgency to conserve these buildings. Therefore, it is of great practical significance to study the impact of coal mining on surface subsidence in the overlap area and its impact on historical buildings, so as to propose technical measures to reduce the impact of coal mining on historical buildings in these areas.

This paper combines the distribution of historical buildings in Shanxi Province, the distribution and mining of coal resources in Shanxi Province and concludes that the overlapping area between coal mining and historical buildings in Shanxi Province is mainly concentrated in Changzhi City, and selects the Lu’an mining area in Changzhi City as the research object, aiming to study the main influencing factors of surface subsidence in the overlapping area by using the gray correlation analysis method. Additionally, this paper aims to investigate the possible influence of surface subsidence on ancient wooden buildings in the overlapping area by combining the surface subsidence in the Lu’an mining area and the formation mechanism, so as to propose technical measures to reduce the influence of surface subsidence caused by coal mining on historical buildings in the overlapping area, and better protect these buildings from damage.

## 2. Historic Buildings and Surface Subsidence Due to Coal Mining in Shanxi

### 2.1. The Number and Distribution of Ancient Wooden Buildings in Shanxi Province

**Figure 1:**
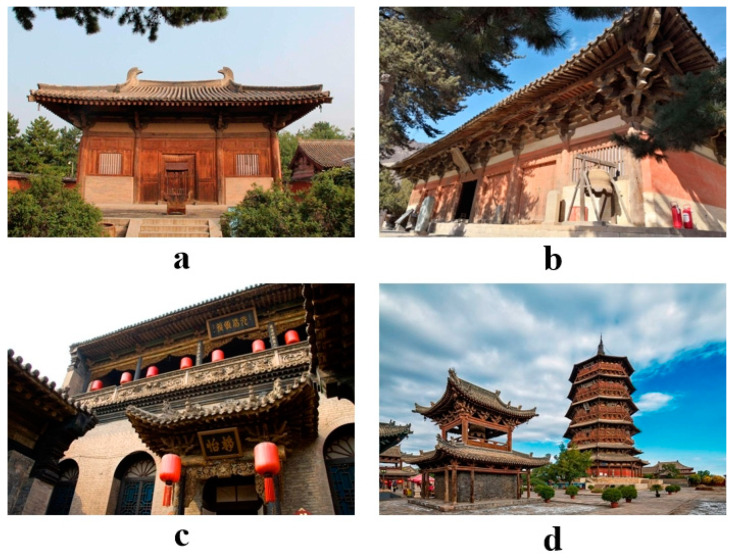
Famous ancient wooden buildings in Shanxi Province.

As one of the most important birthplaces of Chinese civilization, Shanxi has a very long history of architecture. According to the statistics of the relevant departments, there are 263,885 ancient buildings in the country in total. Among them, Shanxi Province has 28,640 registered ancient buildings, occupying 10.85% of the total number of ancient buildings in the country, ranking first in the number of ancient buildings. The number of ancient wooden buildings in Shanxi Province is particularly outstanding; the number of ancient wooden buildings of the Song and Jin periods alone accounted for more than 75% of the total number of ancient wooden buildings [27]. Among them, a few of the most famous ancient buildings are Nanzen Temple in Wutai County, Yizhou City (Figure 1a) and Foguang Temple (Figure 1b), Qiao Family Courtyard in Jinzhong (Figure 1c) and the Wooden Pagoda in Ying County, Shuozhou City (Figure 1d). Shanxi Province only accounts for 1.6% of the country’s land area but has 10.85% of the country’s ancient buildings, so it is easily recognized that “the ground cultural relics look at Shanxi”.

Considering the regional nature of the technology, methods and cultural characteristics of wooden ancient architecture in Shanxi, it is roughly divided into four regions [28]:

(1) Northern region: Yizhou, Datong, Shuozhou.
(2) Central region: Taiyuan, Lyuliang, Jinzhong and Yangquan.
(3) Southeastern region: Changzhi, Jincheng.
(4) Southern region: Linfen, Yuncheng.

**Figure 2:**
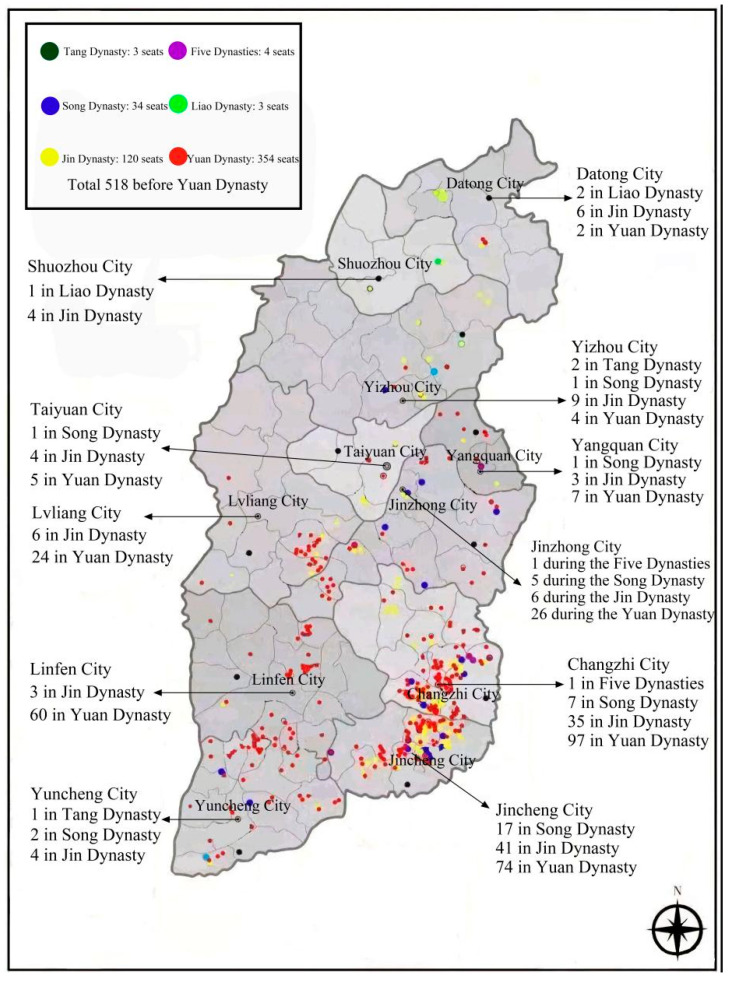
Distribution of ancient wooden buildings in Shanxi Province.

In recent years, the survey shows that 518 ancient wooden buildings exist in Shanxi from before the Yuan Dynasty and during the Yuan Dynasty, accounting for more than 80% of the total number of ancient wooden buildings existing during the Yuan Dynasty and before the Yuan Dynasty, with direct or indirect dating of 244. Among them, Shanxi maintains three ancient wooden buildings of the Tang Dynasty, while the country’s existing ancient wooden buildings of the Tang Dynasty number only four; Shanxi maintains 164 ancient wooden buildings of the Song, Liao and Jin dynasties and before, accounting for approximately 75% of the total amount of these buildings. The number of ancient wooden buildings distributed in the four regions is shown in Figure 2. Among them, the wooden ancient building distribution of Jincheng City and Changzhi City from during the Yuan and before the Yuan dynasty compared to other cities is 132 and 142, respectively, accounting for 25.28% and 27.41% of the province’s existing wooden ancient buildings from this time.

### 2.2. Distribution and Mining of Coal Resources in Shanxi Province

**Figure 3:**
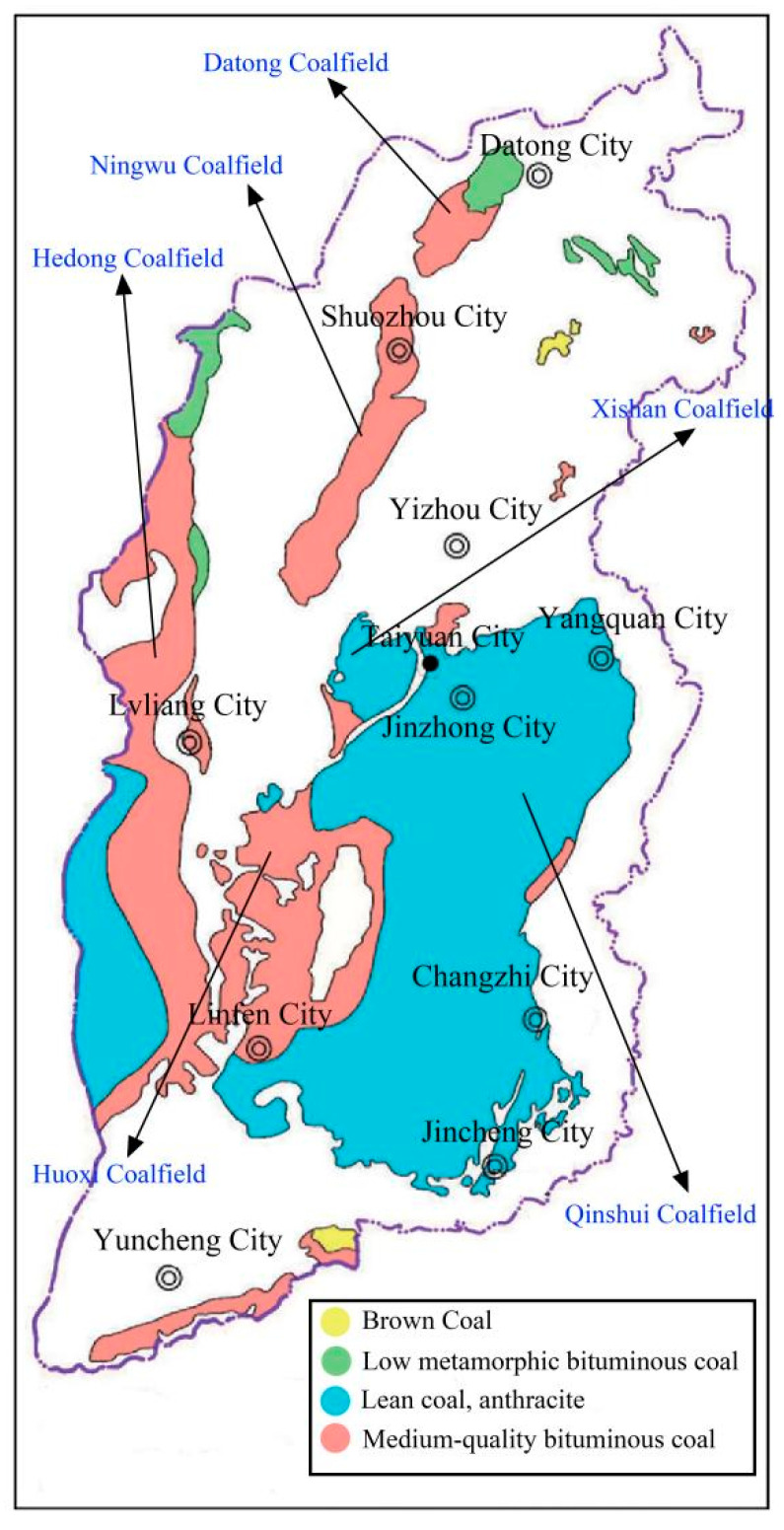
Distribution of coal fields and coal types in Shanxi Province.

Shanxi Province is extremely rich in coal resources, with the highest reserves in China, excellent quality and complete coal types from low metamorphic lignite, long-flame coal to high metamorphic poor coal, anthracite, which covers the entire metamorphic process. According to the data of the Shanxi Bureau of Statistics, the raw coal production of Shanxi exceeded 1 billion tons in 2021, reaching an unprecedented 1.193 billion tons, accounting for 29.31% of the total raw coal production in the country, up 10.5% year-on-year, firmly sitting on the throne of the major domestic coal province. Robust resource reserves are the key foundation for the development of the coal industry in Shanxi, which currently has coal reserves of 270.901 billion tons, ranking first in the country. As one of the world’s five major coal production areas and a critical coal base in China, Shanxi Province contains coal in an area of 64,800 square kilometers, accounting for approximately 40% of the province’s total land area. There are 94 counties and districts in the province storing coal resources, and 91 counties have coal mines. Coal resources are mainly distributed in Datong, Ningwu, Hedong, Xishan, Qinshui, Huoxi (six coal fields) [29] and Hunyuan, Fanchi, Wutai, Qqu, Rui Cheng, Pinglu and other counties (urban areas), as shown in Figure 3.

**Figure 4:**
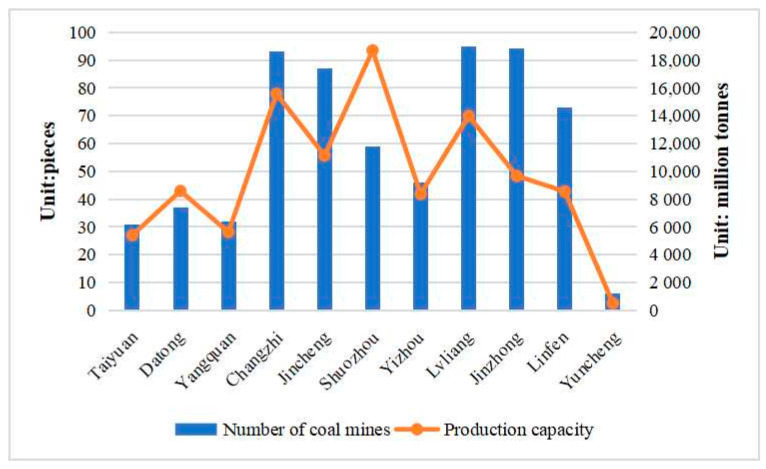
Number and capacity of producing coal mines in Shanxi Province.

According to the announcement of the Shanxi Energy Bureau, as of the end of February 2022, there were 653 coal-producing mines in Shanxi with a combined capacity of 106.4 million t/a. There were 324 medium-sized coal mines with a combined capacity of 259.1 million t/a, accounting for 24.43% of the total coal mine capacity, and 329 large coal mines with a combined capacity of 801.3 million t/a, accounting for 75.57% of the total coal mine capacity, indicating that the current production of coal mines in Shanxi Province is dominated by large coal mines, as shown in Figure 4. Among them, Lyuliang, Jincheng and Changzhi have more coal mines compared to other cities, with 95, 94 and 93 coal mines, accounting for 14.5%, 14.3% and 14.2% of the total coal mines in the province, respectively; Shuozhou has the largest coal mine capacity, with a total capacity of 186.9 million t/a in the city, accounting for 17.6% of the total coal mine capacity in the province.

**Figure 5:**
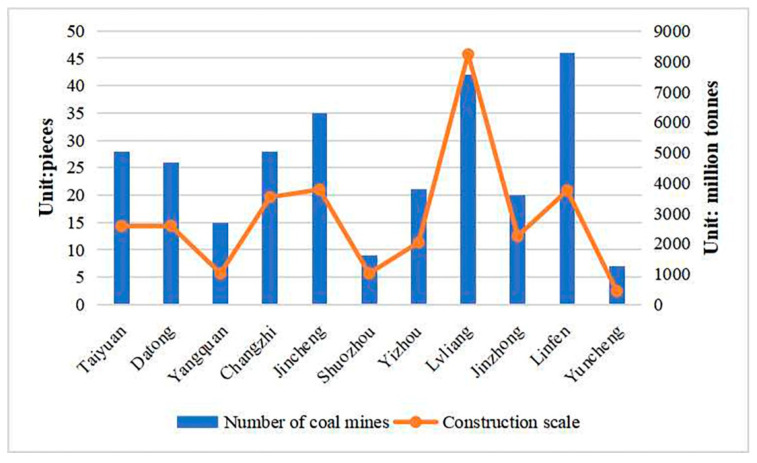
Number of coal mines under construction and construction scale in Shanxi Province.

According to the national coal production capacity (construction coal mines) announcement released by the National Energy Administration, as of 31 December 2018, there were 277 construction coal mines in Shanxi Province, with a total coal mine construction scale of 312.25 million t/a [30]. Among the 277 construction coal mines in Shanxi, there were 5 small coal mines, with a total construction scale of 1.5 million t/a, accounting for 0.48% of the total coal mine construction scale in the province, 193 medium-sized coal mines with a total construction scale of 141.45 million t/a, accounting for 45.30% of the total construction scale of coal mines in the province and 79 large coal mines with a total construction scale of 169.3 million t/a, accounting for 54.22% of the total construction scale of coal mines in the province. The data show that the coal mines built in Shanxi Province in recent years are mainly medium-sized and large coal mines, as shown in Figure 5. The number of coal mines built in Lyuliang and Linfen is higher, with 46 and 42 mines accounting for 16.61% and 15.16% of the total number of coal mines built in the province, respectively. Lyuliang has the largest coal mine construction scale, with a total construction scale of 82.3 million t/a, accounting for 26.36% of the total coal mine construction scale in the province.

### 2.3. Surface Subsidence in Lu’an Mining Area

**Figure 6:**
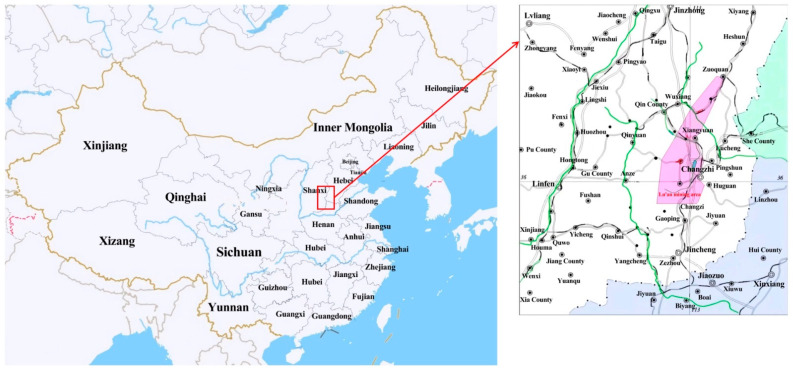
Location Map of Lu’an Mining Area.

The Lu’an mining area is located in the southeast of the Qinshui coalfield in Shanxi Province [31], west of the middle section of the Taihang Mountains, west of the Shangdang Basin, and belongs to the Changzhi Basin, as shown in Figure 6. The mining area is 74.6km long from north to south, and 63.1 km wide from east to west, covering an area of 3044.65 km^2^ [32]; the coal-bearing strata in the area are mainly the Taiyuan Group and Shanxi Group, mainly a set of coal-bearing clastic rock sedimentary construction formed on the basis of the weathering crust of Ordovician limestone [33], with a total stratigraphic thickness of 74.5~306.9 m, generally containing 8~16 layers of coal, with a total thickness of 13.88m and a coal-bearing coefficient of 8.1%.

Combining the actual situation of the Lu’an mining area, coal seam characteristics, coal production capacity and mining situation, this paper investigates the surface subsidence caused by coal mining in the overlapping area based on the existing surface subsidence data processed by SBAS-InSAR technology in the Lu’an mining area and other surface subsidence-related data and obtains the following conclusions.

(1) Due to long-term coal mining in the mining area, the surface subsidence area reaches 79.01 km^2^, accounting for 2.15% of the total mining area, of which the stable subsidence area is 57.5 km^2^, the unstable subsidence area is 21.51 km^2^, and the maximum subsidence of the subsidence area reaches 4.99 m [34].
(2) The areas with significant surface subsidence in the Lu’an mining area are mainly located in the eastern and northern areas of Changzhi County, the southern part of Xiangqi County and the eastern part of Tunliu County.
(3) During 2006.04–2010.05, the southern part of Xiangqi County sinks at an average rate of 0.43 cm per year, Xia Dian at an average rate of 0.16 cm per year, the eastern part of Changzhi County and the northern part of Changzhi County sinks at an average rate of 2.53 cm per year and the eastern part of Tunliu County sinks at an average rate of 3.2 cm.
(4) The distribution of these subsidence areas in the Lu’an mining area is more concentrated. Since 2007, the subsidence rate in the southern part of Xiangqi County has increased slowly, but the subsidence area tends to expand significantly. The subsidence area in the eastern part of Tunliu County does not expand significantly, but the subsidence magnitude increases significantly. The subsidence rate in the eastern part of Changzhi County and the northern part of Changzhi County not only increases in speed, but also the area of the subsidence area continues to expand; in contrast, the subsidence rate and the subsidence area in the Xia Dian area are more stable.
(5) Although the area and scale of subsidence zones formed by other small and medium-sized coal mines in the Lu’an mining area from 2007 onward are smaller, they are almost connected, and their subsidence rates and subsidence areas tend to decrease gradually.

### 2.4. Mechanism of Surface Subsidence Formation in Lu’an Mining Area

(1) Significant local subsidence caused by the change of rock stress field in the mining area

When the underground mining coal seam, the rock support in the roadway and the mining area is only temporary, a mining area will be formed inside the rock body, which will change the stress state of the rock body, cause the redistribution of stress and finally destroy the relative equilibrium state of the rock body. The rock layer at the top of the hollow mining area will slowly produce a downward bending and moving tendency under the action of its gravity and the overlying rock body. When its internal tensile stress exceeds the tensile strength limit of the rock layer, the top plate will first fracture, break and inbreak down, and then three obvious zones will be formed in the overlying rock and soil body of the mining area, namely caving zone, fracture zone and bending deformation zone. Additionally, the top rock layer will move and bend slowly in the form of a beam or cantilever beam bending along the normal direction of the laminae surface, and then produce fracture and delamination until it reaches a new balance. With the expansion of the mining area, the scope of the extraction zone will also be expanded, resulting in the movement of rock layers gradually rising to the surface, and finally forming a large-scale ground subsidence at the surface of the mine.

(2) Large-scale ground subsidence caused by groundwater extraction

The main reason for local surface subsidence within the coal mining area is the destruction of the relative equilibrium state of rock stress, while the large-scale ground subsidence within the coal mining area is the result of the continuous extrusion of the aquifer caused by the massive evacuation of groundwater during the coal mining process [35]. The pumping of groundwater in the coal seam during coal mining will disrupt the equilibrium state of the underground flow field, causing a sharp drop in the groundwater level and finally leading to widespread surface subsidence in the mining area.

(3) Regional faults aggravate surface subsidence

Surface subsidence in coal mining areas originates from mining, but its formation and development are influenced by the intrinsic structure and characteristics of the tectonic environment. Faulting can seriously damage the stress balance state and geological structure of rock strata [36], especially when the fault dip angle is greater than 20° and the drop height is greater than 10 m. Under faulting, the ground stress is in a relatively concentrated state, and the stability of the mine crust will be greatly reduced, resulting in the rocks being extremely fragile and eventually surface subsidence and ground cracks being easily produced.

## 3. Methods

There are many factors influencing surface subsidence in the overburdened area, among which geological factors and mining factors play a major role. Geological factors play a major role in controlling surface subsidence in the coal mining area, and the geological factors affecting surface subsidence in the coal mining area mainly include overburden structure, physical and mechanical properties of overburden, coal seam inclination, Loose Strata properties, tectonic environment, etc. [37,38,39]. Mining factors play an induced role in surface subsidence in coal mining areas, and the formation of the mining area is the determining factor of surface subsidence, and the state of coal resources and coal mining methods directly affect the type and characteristics of surface subsidence; mining factors mainly include mining height, average mining depth, mining size, mining process and roof control methods.

Combining the current coal production capacity, the number of coal mines and the number of ancient wooden buildings in each prefecture-level city in Shanxi Province, it can be seen that the overlapping area between coal mining and ancient wooden buildings in Shanxi Province is mainly concentrated in Changzhi City. Therefore, this paper selects the Lu’an mining area as the research object and uses the gray correlation analysis method to analyze the influencing factors of surface subsidence due to coal mining in the overlapping area and combines the geological conditions and mining conditions of the research area to filter out seven influencing factors, as shown in Table 1.

The mining subsidence coefficient *q* is the ratio of the maximum mining subsidence value *W_cm_* to the projected length of the coal seam normal mining thickness *M* in the plumb direction at the time of full mining (when the length and width of the mining area are quite large). Its size mainly depends on the nature of the overlying rock layer, the thickness of the Loose Strata, the size of the working face, the average mining depth, the number of mining operations and the roof control methods. In other words, under adequate mining conditions, there is a relationship between the surface subsidence coefficient *q* and the geological mining conditions as follows:

$$q=\frac{W_{cm}}{M\times COS\alpha }=f\left[\frac{D_{2}}{D_{1}},H_{0}\right]$$

Additionally, the surface subsidence coefficient *q* as a function of geological mining conditions under non-mining conditions:

$$q=\frac{W_{fm}}{M\times COS\alpha }=f\left[\frac{D_{0}}{H_{0}},\frac{D_{2}}{D_{1}},H_{0}\right]$$

Among them,

*W_cm_*—the maximum subsidence value under full mining conditions, mm;
*W_fm_*—the maximum subsidence value under non-sufficient mining conditions, mm;
*M*—mining thickness, mm;
*D*_2_—thickness of Loose Strata, m;
*D*_1_—thickness of bedrock, m;
*H*_0_—average mining depth of the working face, m;
*D*_0_—working face slope length, length of the projection to the surface by angle, m;
*α*—dip angle of coal seam, degrees.

The surface subsidence coefficient is an important parameter to characterize the intensity of mining subsidence and is an important basis for measuring the surface subsidence of a region. Research shows that the nature of the overlying rock layer is closely related to the surface subsidence coefficient; the harder the overlying rock, the smaller the surface subsidence coefficient, and vice versa; the greater the mining depth and the smaller the mining thickness, the smaller the surface subsidence coefficient. The greater the thickness of the Loose Strata, the greater the mining subsidence coefficient; the roof control method plays a crucial role in surface subsidence, and the mining subsidence coefficient varies greatly with different roof control methods, such as the mining subsidence coefficient is very small when water–sand filling is used; the strike length and tendency length of the working face reflect the degree of mining in two directions, and the mining subsidence coefficient increases with the increase in the degree of mining when it is not fully mined, while the mining subsidence coefficient does not change with the size of the working face when it is fully mined.

The greater the surface subsidence coefficient of an area, the greater the surface subsidence in the area, and, thus, the greater the damage to the stability of the ground building structure; the smaller the surface subsidence coefficient of the area, the less the surface subsidence in the area, and, thus, the less the damage to the stability of the ground building structure.

In this paper, we reviewed and collected the geological condition parameters, mining parameters and observation data of a total of 7 working faces in Wuyang, Wangzhuang, Zhangcun, Sima and Tunliu coal mines in the Lu’an mining area [40]. After careful consideration, we used the surface subsidence coefficient, which characterizes the intensity of mining subsidence, as the reference sequence A_0_, and obtained the data matrix of gray correlation analysis by using the screened 7 factors as the comparison sequence, as shown in Table 2.

The mean value method was used to dimensionlessly process the reference series and comparison series, and the results are shown in Table 3. Then, the absolute value difference between each comparison series and the reference series was calculated to construct the absolute value difference matrix, as shown in Table 4.

Then, the gray correlation coefficients of each datum in the comparison series and the corresponding data in the reference series were calculated using the following gray correlation coefficient formula, and the results are shown in Table 5.

$$\xi \left(x_{i}\right)=\frac{\min_{i}\min_{k}\left|x_{0}(k)-x_{i}(k)\right|+\rho \times \max_{i}\max_{k}\left|x_{0}(k)-x_{i}(k)\right|}{\left|x_{0}(k)-x_{i}(k)\right|+\rho g\max_{i}\max_{k}\left|x_{0}(k)-x_{i}(k)\right|}$$

**Figure 7:**
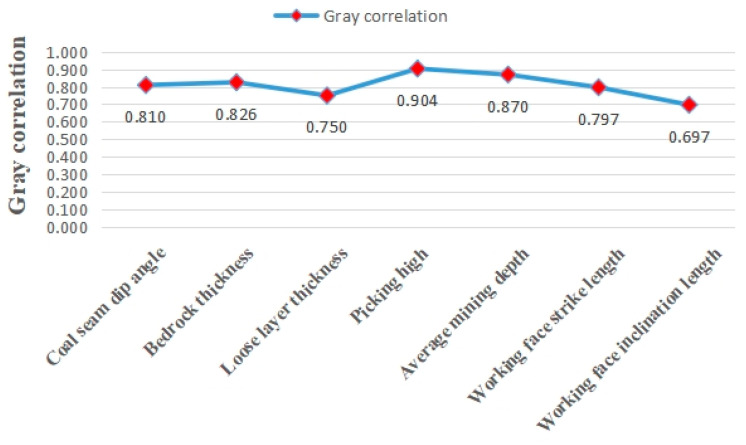
Gray correlation between the influencing factors of surface subsidence and surface subsidence coefficient in Lu’an mining area.

Finally, the average value of the gray correlation coefficients of each sequence was calculated to obtain the gray correlation degree of each influencing factor, as shown in Figure 7. The magnitudes of each influencing factor on the surface subsidence in Lu’an mine are in the order of mining height H > average mining depth H_0_ > bedrock thickness D_1_ > coal seam inclination angle α > working face strike length L_1_ > Loose Strata thickness D_2_ > working face inclination length L.

## 4. Results and Discussion

### 4.1. Analysis of Results

According to the calculation results of gray correlation, the gray correlation of mining height H, average mining depth H0, bedrock thickness D_1_ and coal seam inclination are all greater than 0.800, among which the gray correlation between mining height M and surface subsidence coefficient is the largest at 0.904, the gray correlation between working face inclination length L_2_ and surface subsidence coefficient is the smallest at 0.697 and the average mining depth H_0_ and bedrock thickness D_1_ are the second, indicating that the mechanical properties of overlying rock and coal mining intensity largely determine the degree of surface subsidence in the coal mining area. The mechanical properties of the overburden and the intensity of coal mining largely determine the degree of surface subsidence in the coal mining area; the former reflects the damage resistance of the geological structure of the mine, while the latter reflects the damage ability of human mining activities on the geological environment of the mine, and the resistance between the two is reflected by the degree of surface subsidence [41]. The gray correlation between the average mining depth H_0_, the strike length L_1_ of the working face and the thickness D_1_ of the bedrock and the surface subsidence coefficient are all in the range of 0.750~0.900, and the correlation between the coal seam inclination angle α and the surface subsidence coefficient is more than 0.800, indicating that the surface subsidence in the coal mining area is also closely related to the structure of the overlying rock strata, the size of the coal seam inclination angle itself and the size of the working face. Based on the conclusions of previous scholars, it is not difficult to find that geological factors such as the properties of overlying strata, the thickness of bedrock and the angle of coal seam, and mining factors such as mining height, average mining depth and the size of working face are the main influencing factors controlling the surface subsidence in the coal mining area.

### 4.2. Impacts of Coal Mining on Historic Buildings in Overlapping Areas

**Figure 8:**
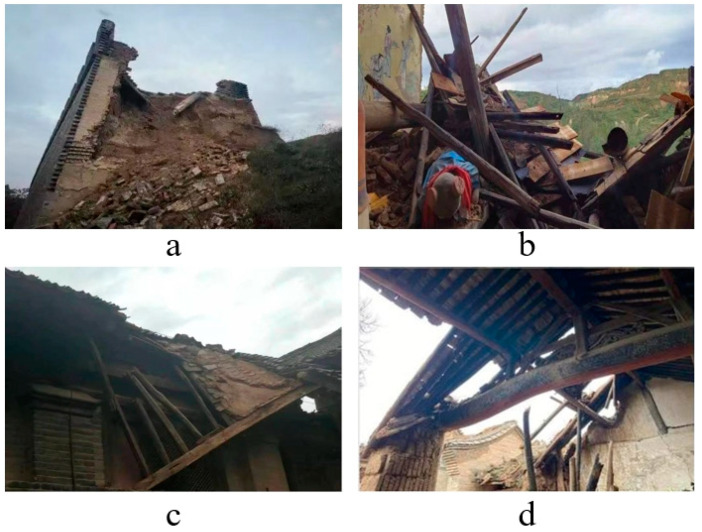
Damage to some ancient wooden buildings in Shanxi.

Surface subsidence can be divided into two types: uniform ground subsidence and uneven ground subsidence. Uniform ground subsidence will make the building sink as a whole and will not have much impact on the stability and use conditions of the building, but when the amount of ground subsidence is large, even if it is uniform subsidence, it may still cause some damage to the building from one aspect. For example, when the surface subsidence is large and the water table is shallow, it will soak the foundation soil of the building with water for a long time or immerse the building in a wet environment for a long time, and then cause serious damage to the building (especially wooden buildings). The uneven ground subsidence often causes the building to produce cracks of different degrees. In this paper, we collected some of the damage to ancient wooden buildings in Shanxi Province. Figure 8a,b show the collapse of ancient wooden buildings caused by ground subsidence in the overlap area, Figure 8c shows the structural damage to ancient wooden buildings caused by ground subsidence in the overlap area, while Figure 8d shows the deformation of ancient wooden building beams caused by ground subsidence in the overlap area. After analysis, the surface subsidence due to coal mining in the overlap area mainly affects the wooden buildings as follows:

(1) Cause the ancient wooden building walls to produce larger cracks. In general, the superstructure of the building has a certain degree of stiffness; after being subjected to load and work together with the foundation, surface subsidence caused by coal mining will often subject the ancient wooden building foundations to varying degrees of subsidence, and the uneven subsidence of the foundation is limited by the flexural stiffness of the superstructure, which will cause the superstructure to produce a certain amount of additional stress, and ultimately produce large cracks in the walls of the building due to the main tensile stress being too strong.

(2) The beam frame of ancient wooden buildings will produce deformation. The overlap area due to coal mining surface subsidence often affects the force structure of ancient buildings, destroying the relative force equilibrium state of the building. In the long run, it will cause the deformation of the beam frame of ancient wooden buildings, and even obvious distortion.

(3) Affect the mortise and tenon structure in ancient wooden buildings, thus destroying the overall structural stability of ancient wooden buildings, indirectly making the ancient wooden buildings tumble, collapse, etc. Under the influence of ground subsidence for a long time, the beam–column connection is prone to the tenon pulling phenomenon, which will reduce the effective stress cross-section of the beam and column members, and the phenomenon of pulling, pressing, bending, shearing and other damage occurs very easily, seriously damaging the overall stability of ancient wooden buildings.

(4) If the wooden building is in a long-term wet environment, and this can cause the wooden building’s internal beams, columns and load-bearing parts to decay, and finally lead to the collapse of the wooden building with the passing of time or the advent of natural disasters (such as earthquakes, rainstorms, etc.). When the overlap area surface subsidence is large, with a shallow water table and rising diving level, it will subject the overlap area’s ancient wooden buildings as a whole to a humid environment.

(5) Directly make the ancient wooden buildings collapse. Coal mining will often form a hollow area; with the expansion of the hollow area, the surface subsidence caused by coal mining will also become larger; it will cause the foundation of ancient wooden buildings to collapse, and finally lead to the collapse of ancient wooden buildings.

(6) It may cause the ancient wooden building as a whole to tilt. When uneven subsidence occurs in the overlap area, the foundation of the ancient wooden building will also be subject to different degrees of subsidence, which will destroy the overall structural stability of the ancient wooden building and finally lead to the tilting of the ancient wooden building as a whole.

### 4.3. Technical Measures to Reduce the Impact of Surface Subsidence on Historic Buildings in Overlapping Areas

According to the influence of surface subsidence in coal mining areas on ancient wooden buildings and the main influencing factors of surface subsidence in the coal mining area, we can take the following measures to reduce the surface subsidence due to coal mining in overlapping areas, so as to reduce its influence on historical buildings.

(1) Use of the infill coal mining method to deal with the overburdened area. When using the infill coal mining method, the degree of damage to the overlying rock is relatively small, which can not only moderate the mineral pressure generated by the working face support pressure, but also effectively reduce the degree of surface subsidence in the overlapping area; moreover, the degree of reduction depends on the filling method and filling material, thus reducing the damage to the foundation and structural stability of ancient wooden buildings caused by mining subsidence in the overlapping area.

(2) Adopt the room and pillar coal mining method. This coal mining method can effectively prevent surface subsidence caused by coal mining, and has better economic applicability, but the recovery rate of mining is lower.

(3) Adopting the strip-mining method. The strip-mining method is to divide the mined coal seam into a more formal strip shape for mining, mining one, leaving one, and using the strip coal column left unmined to bear all the load of the overlying rock seam [42], in order to achieve the purpose of reducing the surface subsidence in the overlapping area, thereby reducing its impact on ancient wooden buildings.

(4) Adopt high-pressure grouting for the off-set zone of the mining area. After underground coal mining, due to the different subsidence speed of the overlying rock layer, there will be cracks between the rock layers; in case of this situation, the coal seam’s overlying rock layer can be mined, with drilling in the off-layer zone and mud injected into the gap, which will slow down the sinking speed of the surface to a certain extent, indirectly reducing the overlap area due to coal mining and reducing the impact of surface subsidence on ancient wooden buildings.

(5) Intermittent mining for thick coal seams with horizontal or inclined stratification can, to a certain extent, reduce the damage to the overlying rock strata, which in turn can reduce the surface subsidence caused by coal mining in the overlap area [43], and finally indirectly reduce its impact on ancient wooden buildings.

(6) Reasonable setting of coal mining height and working face length in the overlap area.

## 5. Conclusions

This paper introduces the distribution of historical buildings in Shanxi Province and the distribution and mining of coal resources in Shanxi Province by collecting data, and then combines the current coal production capacity, the number of coal mines and the number of ancient wooden buildings in each prefecture-level city in Shanxi Province to conclude that the overlapping area of coal mining and ancient wooden buildings in Shanxi Province is mainly concentrated in Changzhi City. We then selected the Lu’an mining area in Changzhi City, Shanxi Province, as the study area, and used the gray correlation analysis method to take the surface subsidence coefficient, which characterizes the intensity of mining subsidence, as the reference sequence and screened seven factors as the comparison sequence. Following this, the gray correlation between each influencing factor and the surface subsidence coefficient of the reference sequence was calculated to obtain that geological factors such as the nature of overlying rock strata, bedrock thickness and dip angle of coal seam, and mining factors such as mining height, average mining depth and working face size, largely determine the extent of surface subsidence in the overlapping area.

Based on the existing surface subsidence in the Lu’an mining area and the formation mechanism, we investigated the effects of surface subsidence caused by coal mining in the overlapping area on ancient wooden buildings: (1) causing large cracks in the walls of ancient wooden buildings; (2) deforming the beams of ancient wooden buildings; (3) affecting the mortise and tenon structure of ancient wooden buildings, thus destroying the overall structural stability of ancient wooden buildings and indirectly causing ancient wooden buildings to tumble, collapse, etc.; (4) subjecting the ancient wooden buildings to a long-term humid environment, which in turn causes the ancient wooden buildings’ internal beams, columns and load-bearing parts to decay, and finally leads to the collapse of ancient wooden buildings with the passing of time or the advent of natural disasters (such as earthquakes, heavy rain, etc.). When the amount of surface subsidence in the overlap area is large, the groundwater level is shallow and the dive level rises, which subjects all ancient wooden buildings in the overlap area to a humid environment; (5) directly making the ancient wooden buildings collapse; (6) possibly making the ancient wooden buildings as a whole tilt.

Finally, combining the main influencing factors of surface subsidence in the overlap area and the influence on ancient wooden buildings, technical measures are proposed to reduce the influence of surface subsidence on historical buildings due to coal mining in the overlap area: (1) using the method of filling coal mining to deal with the empty area; (2) adopting the method of room and pillar coal mining; (3) adopting the method of strip coal mining; (4) adopting high-pressure grouting for the off-seam zone in the empty area; (5) intermittent mining for thick coal seams showing horizontal or inclined stratification; (6) reasonably set the coal mining height in the overlapping area and the working face length.

## Figures and Tables

**Table 1:** Influence factors of surface subsidence caused by coal mining in Lu’an mining area.

Macro Factors	Number	Influencing Factors
Geological factors	A_1_	Dip angle of coal seam α/°
	A_2_	Bedrock thickness D_1_/m
	A_3_	Loose Strata thickness D_2_/m
Mining Factors	A_4_	Mining height H/m
	A_5_	Average mining depth H_0_/m
	A_6_	Workface strike length L_1_/m
	A_7_	Working surface inclination length L_2_/m

**Table 2:** Data on factors influencing surface subsidence in Lu’an mining area.

Number	A_0_	A_1_	A_2_	A_3_	A_4_	A_5_	A_6_	A_7_
1	0.72	8	187	25	3	212	770	180
2	0.72	7	144	20	3	164	500	160
3	0.80	8	510	40	6.5	540	1514	190
4	0.82	7	300	29	5.4	329	1450	200
5	0.82	2	288	62	6.2	350	223	1560
6	0.86	4	288	30	6.2	318	716	188
7	0.94	5	56	186	6.7	242	690	174

**Table 3:** Data on factors influencing surface subsidence in Lu’an mine after dimensionless treatment.

Number	A_0_	A_1_	A_2_	A_3_	A_4_	A_5_	A_6_	A_7_
1	0.8873	1.3659	0.7383	0.4464	0.5676	0.6886	0.9193	0.4751
2	0.8873	1.1951	0.5685	0.3571	0.5676	0.5327	0.5970	0.4223
3	0.9859	1.3659	2.0135	0.7143	1.2297	1.7541	1.8076	0.5015
4	1.0106	1.1951	1.1844	0.5179	1.0216	1.0687	1.7312	0.5279
5	1.0106	0.3415	1.1371	1.1607	1.1730	1.1369	0.2662	4.1176
6	1.0599	0.6829	1.1371	0.5357	1.1730	1.0329	0.8549	0.49621
7	1.1585	0.8537	0.2211	3.3214	1.2676	0.7861	0.8238	0.4592

**Table 4:** Absolute value difference data of surface subsidence influencing factors in Lu’an mining area.

|A_0_-A_1_|	|A_0_-A_2_|	|A_0_-A_3_|	|A_0_-A_4_|	|A_0_-A_5_|	|A_0_-A_6_|	|A_0_-A_7_|
0.4786	0.1490	0.4409	0.3197	0.1987	0.0320	0.4122
0.3078	0.3188	0.5302	0.3197	0.3546	0.2903	0.4650
0.3800	1.0276	0.2716	0.2438	0.7682	0.8217	0.4844
0.1845	0.1738	0.4927	0.0110	0.0581	0.7206	0.4827
0.6691	0.1265	0.1501	0.1624	0.1263	0.7444	3.1070
03770	0.0772	0.5242	0.1131	0.0270	0.2050	0.5637
0.3048	0.9374	2.1629	0.1091	0.3724	0.3347	0.6993

**Table 5:** Gray correlation coefficients of surface subsidence influencing factors and surface subsidence coefficients in Lu’an mining area.

Number	A_1_	A_2_	A_3_	A_4_	A_5_	A_6_	A_7_
Number of grey contacts	0.7699	0.9189	0.7844	0.8352	0.8928	0.9868	0.7959
	0.8405	0.8356	0.7508	0.8352	0.8199	0.8485	0.7751
	0.8092	0.6061	0.8572	0.8705	0.6739	0.6587	0.7677
	0.9002	0.9057	0.7646	1.0000	0.9708	0.6880	0.7683
	0.7039	0.9313	0.9183	0.9118	0.9314	0.6808	0.3357
	0.8104	0.9594	0.7530	0.9387	0.9899	0.8897	0.7389
	0.8419	0.6281	0.4210	0.9410	0.8123	0.8286	0.6945

## Data Availability

Some or all data that support the findings of this study are available from the corresponding author upon reasonable request.

## References

[1] Li L., YLei L., Wu S.M., He C.Y., Yan D. (2018). Study on the Coordinated Development of Economy, Environment and Re-source in Coal-Based Areas in Shanxi Province in China: Based on the Multi-Objective Optimization Model. Resour. Policy.

[2] Xue J.J. (2020). Ground Surface Subsidence and Ecological Disturbance in the Mining Area of Loess Plateau with Complex Environments: A Case Study from Xuangang Coal Mining Subsidence Area. Ph.D. Thesis.

[3] Chen F., Lin H., Zhang Y., Lu Z. (2012). Ground subsidence geo-hazards induced by rapid urbanization: Implications from InSAR observation and geological analysis. Nat. Hazards Earth Syst. Sci..

[4] Dong S., Samsonov S., Yin H., Ye S., Cao Y. (2014). Time-series analysis of subsidence associated with rapid urbanization in Shanghai, China measured with SBAS InSAR method. Environ. Earth Sci..

[5] Saleh M., Becker M. (2019). New estimation of Nile Delta subsidence rates from InSAR and GPS analysis. Environ. Earth Sci..

[6] Zebker H.A., Goldstein R.M. (1986). Topographic mapping from interferometric synthetic aperture radar observations. J. Geophys. Res. Solid Earth.

[7] Zhang L., Ding X., Lu Z., Jung H., Hu J., Feng G. (2013). A novel multitemporal InSAR model for joint estimation of defor-mation rates and orbital errors. IEEE Trans. Geosci. Remote Sens..

[8] Tang L., Hou H., Zhang S., Guo S., Mi J. (2021). Monitoring of short revisited SAR data in rapid deformation field of mining area. Sci. Surv. Mapp..

[9] Giardina G., Hendriks M., Rots J.G. Numerical Analysis of Tunnelling Effects on Masonry Buildings: The Influence of Tunnel Location on Damage Assessment. Proceedings of the 7th International Conference on Structural Analysis of Historic Constructions.

[10] Johnson K.S. (2004). Subsidence hazards due to evaporite dissolution in the United States. Environ. Geol..

[11] Lee D.-K., Mojtabai N., Lee H.-B., Song W.-K. (2013). Assessment of the influencing factors on subsidence at abandoned coal mines in South Korea. Environ. Earth Sci..

[12] Solarski M., Machowski R., Rzetala M., Rzetala M.A. (2022). Hypsometric changes in urban areas resulting from multiple years of mining activity. Sci. Rep..

[13] Marschalko M., Yilmaz I., Křístková V., Fuka M., Kubečka K., Bouchal T. (2013). An indicative method for determination of the most hazardous changes in slopes of the subsidence basins in underground coal mining area in Ostrava (Czech Republic). Environ. Monit. Assess..

[14] Dang V.K., Nguyen T.D., Dao N.H., Duong T.L., Dinh X.V., Weber C. (2021). Land subsidence induced by underground coal mining at Quang Ninh, Vietnam: Persistent scatterer inter-ferometric synthetic aperture radar observation using Sentinel-1 data. Int. J. Remote Sens..

[15] Can E., Kuscu S., Kartal M.E. (2012). Effects of mining subsidence on masonry buildings in Zonguldak hard coal region in Turkey. Environ. Earth Sci..

[16] Zhang Q., Cheng H., Gui Y., Cao J. Foundation Pit Excavation on Surrounding Buildings Effect Analysis. Proceedings of the 2nd International Conference on Mechatronics and Applied Mechanics (ICMAM2012).

[17] Unlu T., Akcin H., Yilmaz O. (2013). An integrated approach for the prediction of subsidence for coal mining basins. Eng. Geol..

[18] Ma X.T., Bao C., Shang H., Wang H., Doh S., Hu J., Shu H. (2022). Structural response of RC frame under surface curvature and differential settlement in mining areas. Phys. Chem. Earth.

[19] Zhou J.J. (2017). Study on Surface Subsidence Caused by Rail Transit Construction and Its Influence on Ancient Buildings. Master’s Thesis.

[20] Song S.J.X., Zhao G., Xie J., Guan Y.Y. Grey Correlation Analysis and Regression Estimation of Mining Subsidence in Yu-Shen-Fu Mining Area. Proceedings of the 3rd International Conference on Environmental Science and Information Application Technology (ESIAT).

[21] Zhang M., He H., Jin X., Qu Y., Guo H. (2021). Research on Key Factors Influencing Surface Subsidence of Paste Backfilling Mining in Thick Coal Seam of Deep Mine. Adv. Civ. Eng..

[22] Luan Y., Ji Z., Cui Z., Liang Y. (2022). Prediction and analysis of surface subsidence coefficient based on combined weight. Coal Sci. Technol..

[23] Zhu X.X., Zhang W., Wang Z., Wang C., Li W., Wang C.H. (2020). Simulation Analysis of Influencing Factors of Subsidence Based on Mining under Huge Loose Strata: A Case Study of Heze Mining Area, China. Geofluids.

[24] Huang C.L. (2013). Study of Impacts to Geological Environments by Coal Mining Activites in Lu’an Mining Area. Ph.D. Thesis.

[25] Gang C., Qiong W., Ming L.X. (2020). Prediction of Surface Subsidence Due to Mining. Integr. Ferroelectr..

[26] Du Y.H., Yang W.F. (2022). Construction of investigation and monitoring system of coal mining subsidence area based on multi-source data in Shanxi province. Surv. Mapp. Bull..

[27] Chai L., Zhu X.D., Wang C.E. Analysis of Temple Form and Sculpture Value of Song Dynasty in Southeast Shanxi—The Case of Zhangzi Chongqing Temple. Proceedings of the 2nd International Conference on Civil Engineering and Transportation (ICCET 2012).

[28] Li H.Z. (2021). Distribution and Regional Characteristics of Wooden-Frame Buildings Before Yuan Dynasty in Shanxi Province. Nat. Cult. Herit. Stud..

[29] Guo Y.J., Zhao X., Chu K.J. (2018). Thought on Development of Clean and Efficient Utilization of Coal Resources in Shanxi. Energy Energy Effic..

[30] Huo C. (2020). Study on Coal Resources Distribution Features and Exploration, Exploitation Layout in Shanxi Province. Coal Geol. China.

[31] Tian L., Cao Y., Chai X., Liu T., Feng P., Feng H., Zhou D., Shi B., Oestreich R., Rodvelt G. (2015). Best practices for the determination of low-pressure/permeability coalbed methane reservoirs, Yuwu Coal Mine, Luan mining area, China. Fuel.

[32] Pan J.E.A., Sun T., Hou Q., Cao Y., Deng X.W. (2015). Examination of the formation phases of coalbed methane reservoirs in the Lu’an mining area (China) based on a fluid inclusion analysis and Ro method. J. Nat. Gas Sci. Eng..

[33] Zhang M., He H., Zhang Y., Jin X., Liang X., Zhang Y., Guo H. (2020). Research on the Deformation and Control Technology of Surrounding Rock in Entry Retaining along the Gob Side. Adv. Civ. Eng..

[34] Tang X.H. (2016). Current Situation, Hazards and Management of Coal Mining Subsidence Area in Shanxi. Ecol. Econ..

[35] Gong X., Geng J., Sun Q., Gu C., Zhang W. (2020). Experimental study on pumping-induced land subsidence and earth fissures: A case study in the Su-Xi-Chang region, China. Bull. Eng. Geol. Environ..

[36] Yang C.-S., Zhang Q., Zhao C.-Y., Wang Q.-L., Ji L.-Y. (2014). Monitoring land subsidence and fault deformation using the small baseline subset InSAR technique: A case study in the Datong Basin, China. J. Geodyn..

[37] Choi J.K., Kim K., Lee S., Won J.S. (2010). Application of a fuzzy operator to susceptibility estimations of coal mine subsidence in Taebaek City, Korea. Environ. Earth Sci..

[38] Oh H.-J., Ahn S.-C., Choi J.-K., Lee S. (2011). Sensitivity analysis for the GIS-based mapping of the ground subsidence hazard near abandoned underground coal mines. Environ. Earth Sci..

[39] Saro L., Hyun-Joo O., Ki-Dong K. (2010). Statistical Spatial Modeling of Ground Subsidence Hazard near an Abandoned Underground Coal Mine. Disaster Adv..

[40] Hu H.F. (2012). Research on Surface Subsidence Regularity and Prediction under Composite Mediums with Different Thickness Rations of Loose and Bedrock. Ph.D. Thesis.

[41] Liu D.H., Deng N., Yao T. (2020). Analysis of main controlling factors of coal mining subsidence in Lu’an Mining Area. Min. Saf. Environ. Prot..

[42] Zhu X., Guo G., Liu H., Yang X. (2019). Surface subsidence prediction method of backfill-strip mining in coal mining. Bull. Eng. Geol. Environ..

[43] Zhai M., Bai H., Wu L., Wu G., Yan X., Ma D. (2022). A reinforcement method of floor grouting in high-water pressure working face of coal mines: A case study in Luxi coal mine, North China. Environ. Earth Sci..

